# MStractor: R Workflow Package for Enhancing Metabolomics Data Pre-Processing and Visualization

**DOI:** 10.3390/metabo11080492

**Published:** 2021-07-29

**Authors:** Luca Nicolotti, Jeremy Hack, Markus Herderich, Natoiya Lloyd

**Affiliations:** 1 The Australian Wine Research Institute, Adelaide, SA 5064, Australia; jeremy.hack76@gmail.com (J.H.); markus.herderich@awri.com.au (M.H.); natoiya.lloyd@awri.com.au (N.L.); 2 Metabolomics Australia, The Australian Wine Research Institute, Adelaide, SA 5064, Australia

**Keywords:** metabolomics, data analysis, pre-processing, R programming language, LC/MS

## Abstract

Untargeted metabolomics experiments for characterizing complex biological samples, conducted with chromatography/mass spectrometry technology, generate large datasets containing very complex and highly variable information. Many data-processing options are available, however, both commercial and open-source solutions for data processing have limitations, such as vendor platform exclusivity and/or requiring familiarity with diverse programming languages. Data processing of untargeted metabolite data is a particular problem for laboratories that specialize in non-routine mass spectrometry analysis of diverse sample types across humans, animals, plants, fungi, and microorganisms. Here, we present MStractor, an R workflow package developed to streamline and enhance pre-processing of metabolomics mass spectrometry data and visualization. MStractor combines functions for molecular feature extraction with user-friendly dedicated GUIs for chromatographic and mass spectromerty (MS) parameter input, graphical quality-control outputs, and descriptive statistics. MStractor performance was evaluated through a detailed comparison with XCMS Online. The MStractor package is freely available on GitHub at the MetabolomicsSA repository.

## 1. Introduction

Over the last few decades, mass spectrometry (MS) has become the technique of choice to profile metabolites in biological systems, specifically when untargeted strategies are required for characterizing the complexity of biological samples. Significant improvements to the instrument’s sensitivity resulted in exponential growth of metabolites detected in a single analysis (>10^3^), and faster chromatographic separations allow analysis of large sample sets. As a consequence, a single instrument can, over 24 h, produce data covering 10^5^ to 10^6^ potential metabolites. Hence, data processing has become a critical step that limits throughput, productivity, and potentially also the quality of interpretation of raw mass spectrometry data. Availability of bioinformatics workflows and software that enable reliable data pre-processing, rapid throughput, and production of reliable information is essential for the quality of the analytical results and biological interpretation [1,2,3,4].

Software for processing metabolomics data is in either a proprietary or open-source format [1,5]. There are many solutions available, with various degrees of sophistication; some software supports the entire metabolomics data-processing pipeline, while other programs are specialized for specific tasks such as feature detection, statistical analysis, or metabolite identification. In addition, many tools offer processing solutions limited to single technology platforms such as GC/MS [6] or LC/MS.

Commonly used commercially available software includes Mass Profiler by Agilent Technologies, Bruker Metaboscape, Progenesis QI by Waters, and Compound Discoverer by Thermo Fisher Scientific. On the other hand, a fast-progressing area in metabolomics is the development of freely available and open-source tools for all facets of data processing [7,8]. Popular open-source software such as MetaboAnalyst, MetaboNexus, XCMS, MZmine2, and MAVEN [9,10,11,12,13] provide the community with advanced tools to manage, explore, and annotate the increasing complexity of data generated from new and evolving technologies. Some popular open-source packages and platforms like XCMS and XCMS online also include tools for statistical analysis [14,15,16].

Bioconductor, an open-source platform for bioinformatics analysis based on the R programming language, represents one of the best options available when dealing with both GC/MS and LC/MS datasets. It offers a range of packages covering pre-processing of GC/MS and LC/MS data, reconstruction and annotation of deconvoluted spectra [17], and complex data visualization.

Despite the many advantages of open-source tools, some aspects can be improved further. As an example, XCMS and CAMERA [17] contain hundreds of functions (around 1000 for XCMS) with many different parameter-setting options. This provides experienced users with desirable flexibility and allows the development of optimized data pre-processing solutions. At the same time, this can be a challenge for time-poor researchers, occasional users, technical staff, and laboratories that analyze many diverse biological sample types. In addition, it can be difficult for users who are not familiar with the R language to build a suitable script for processing a specific dataset.

Here we present MStractor, an R workflow package that offers a generic, user-friendly framework for simplifying and automating the supervised analysis of LC/MS data from untargeted metabolomics experiments. MStractor organizes, integrates, and implements a range of functions and provides GUIs for project set-up, quality control (QC) data monitoring, graphical outputs, data normalization, and descriptive statistics calculations. In addition, it provides the operator with guidance for the selection of meaningful parameters related to the analytical platform used, thus minimizing potential parameter input errors. MStractor workflow performance is critically evaluated in the following sections of this paper and compared in detail with XCMS Online [16], which is one of the most popular GUI-based tools for online pre-processing and visualisation of LC/MS metabolomics datasets.

## 2. Discussion and Results

### 2.1. General Overview

The MStractor workflow translates raw mass spectrometry data into a base peak table output that can be used for statistical analysis. It is freely available on Github and combines a range of tools into a seamless workflow for processing large batches of metabolomics datasets quickly and easily.

It is written in the R programming language, and the functionality of the toolbox is user-adaptable, extendable, and practical for wider community use by analysts and biologists. It includes high-performing tools such as XCMS and CAMERA [16,17] with specific parameter-setting functions to minimize processing errors and graphical outputs for QC.

**Figure 1:**
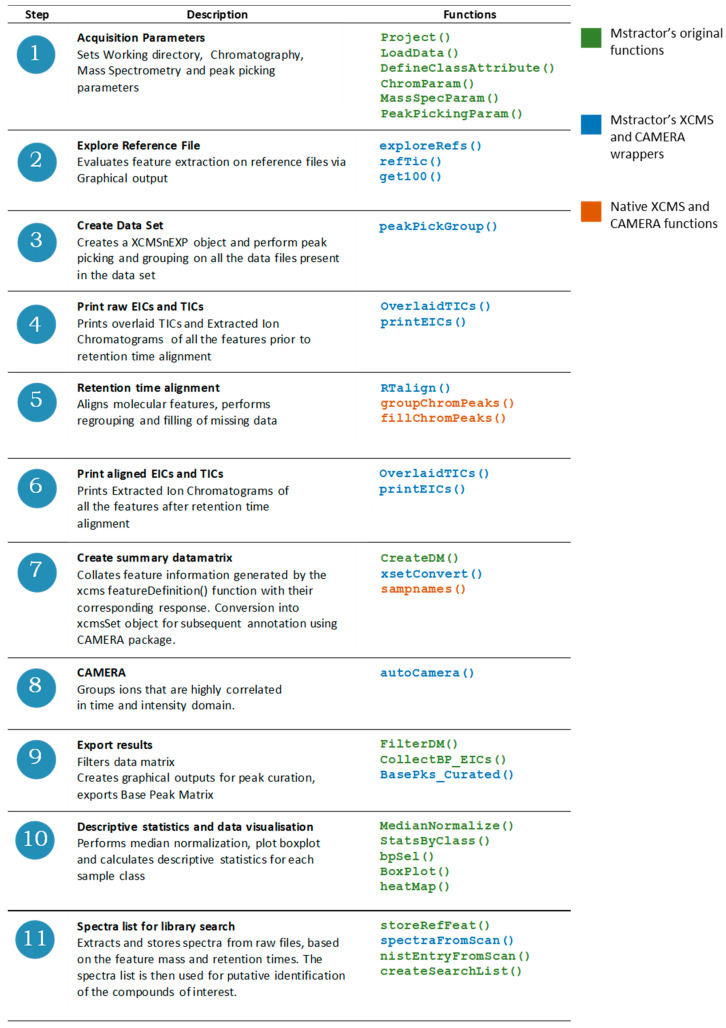
Schematic representation of the MStractor workflow and associated functions.

A schematic representation of the MStractor framework is displayed in Figure 1, in which the different steps of the workflow are displayed together with the associated functions. The workflow has been designed to use XCMS functions compatible with the most recent xcmsEXPn object type. The package also offers the possibility of running the workflow using a lower computing power environment that utilizes the former XCMS object type (xcmsSet). This option is detailed in the package documentation and is not described here in the interest of brevity.

MStractor functions can be classified into three groups:

1. Functions developed by the authors to provide the user with GUIs for parameter input and data QC monitoring (in green in Figure 1).
2. Wrappers of XCMS and CAMERA functions that are implemented with additional code to automate routine operations and graphical output generation (in blue in Figure 1).
3. Native CAMERA and XCMS functions. All the arguments and parameters required by these functions are automatically generated along the workflow (in orange in Figure 1).

### 2.2. MStractor Performance Evaluation

MStractor performance was benchmarked against the existing XCMS Online tool. XCMS Online was chosen for the following reasons: both programs are directed to the same user type; they share core workflow steps (parameter input, peak detection, retention time alignment, and data visualization); and they both rely on XCMS and CAMERA packages for feature extraction and annotation. The LC/MS demo dataset for the software comparison and the chosen parameters are described in the Materials and Methods section.

#### 2.2.1. Data Input

Raw data files are converted into one of the supported file formats (mzXML, mzData, CDF and mzML). Files need to be stored in a directory named “MSfiles”, where raw data files are arranged in different subdirectories according to their class (see package vignettes), and based on this folder structure, each analytical sample is automatically assigned to the class of belonging within the MStractor workflow. XCMS Online (Pairwise option) requires separate uploads of each class of samples.

Both workflows provide similar tools for data upload; XCMS Online uses a drag-and-drop/file explorer option, and MStractor uses the functions Project() and LoadData(). Project() allows setting the working directory via a file explorer GUI, while LoadData() uploads raw data within the R working environment.

Compared to XCMS Online, the data input step in MStractor provides two additional advantages:

1. Project() allows two analytical replicates to be defined that are used in the early steps of the workflow to evaluate peak detection parameters. It also generates a QC directory where all the QC graphical outputs and data tables generated along the intermediate steps are stored.
2. DefineClassAttributes() automatically defines symbols, colours, and identifiers for each sample class. In this way, samples belonging to different classes are labelled with different colours and symbols in the graphical outputs generated. This enables easier interpretations of the generated plots, as well as performing additional quality control of the loaded files.

#### 2.2.2. Data Processing Parameter Input

Parameter input for data processing in MStractor works differently in comparison to XCMS Online.

XCMS Online provides five different tabs for parameter input; “General”, “Feature detection”, “Retention time correction”, “Alignment”, and “Annotation”.

All parameters must be entered before submitting the job. Although input parameters are saved and stored within the project, the user has no option to perform adjustments along the workflow.

Conversely, MStractor provides GUI for input parameters in a stepwise manner, and an example of the GUI is displayed in Figure S2. The advantage of the MStractor stepwise parameter input scheme is to provide QC and flexibility during every stage of processing. The fit for purpose of entered values can be tested, and if required, changed prior to re-executing the functions, as described in Section 2.2.3.

General chromatographic and mass spectrometry settings (such as retention time deviation and polarity) are defined via ChromParam() and MassSpecParam() functions, while feature-detection parameters are entered via PeakPickingParam().

In addition to the options available for peak detection in XCMS Online, MStractor also allows defining values for “integration threshold” and “sensitivity”, which are useful for limiting the number of features detected in the background range, thus minimizing noise in the extracted data.

Feature grouping is defined within peakPickGroup(), while the retention time parameter input is carried out via the RTAlign() function.

Isotopic pattern and adduct annotation in XCMS Online is based on ppm and *m/z* absolute error parameters, which are entered via the “Annotation” tab.

In MStractor, this is achieved via the “CAMERA” package, the main functions of which are grouped within the autoCAMERA() function, which also provides a GUI for parameter input. After parameter input, autoCAMERA()identifies *m/z* ions that are likely to belong to the same molecule and groups them in pseudospectra. After this, annotation of the isotopic pattern and related adducts is performed. Input parameters used for autoCamera() are listed in Table 1.

#### 2.2.3. Functionalities for Workflow QC

The standout feature of MStractor is the generation of quality control (QC) outputs for intermediate (framework) steps, a characteristic that is absent in XCMS Online, as all the outputs are only accessible upon job completion. The most valuable QC features are feature-detection parameter evaluation and feature curation.

**Figure 2:**
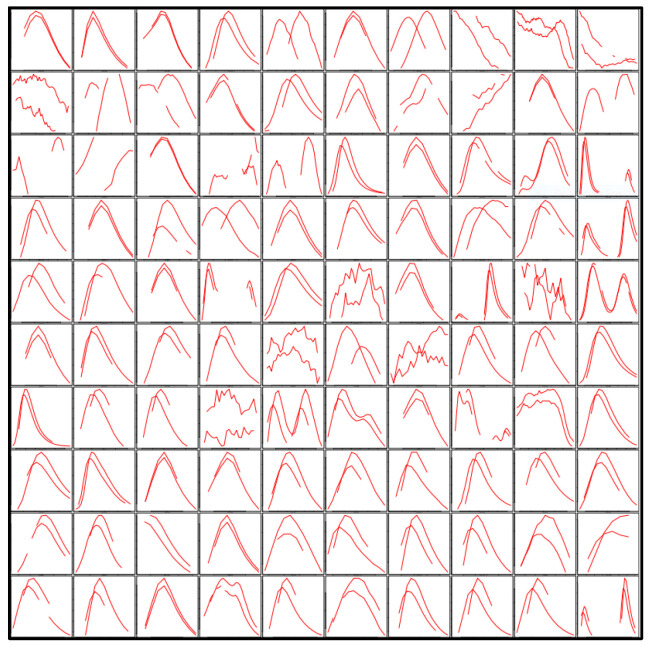
Example of graphical output for input-parameter evaluation.

For feature-detection parameter evaluation, peak-picking parameters are tested on the reference files selected at the beginning of the workflow, via Project(). Using the exploreRef() function, peak detection and grouping is performed on the selected reference files. refTic() prints the total ion chromatogram for reference files, while get100() prints an image of 100 features randomly selected across the retention time range (an example is reported in Figure 2). The plot gives a general overview of the peak-picking process, showing whether extensive background noise is present or if multiple peaks were grouped into a single feature. It is, therefore, possible to adjust the input values until the desired peak-picking quality is achieved, without the need to wait until the entire workflow has been completed.

In respect to feature curation, the CollectBP_EICs() function prints images of the extracted ion chromatograms related to the base peak matrix in two identical folders named “EICs_BasePeaks” and “EICs_BasePeaks_Curated”. The Base Peak matrix output is also stored within the working directory in a tab separated value (.tsv) file named “Pks_BPs”. The “EICs_BasePeak” is used as a backup directory and stores all the EICs prior to curation. The EICs in the directory “EICs_BasePeaks_Curated” need to be inspected by the user, and the images (features) that do not resemble resolved chromatographic peaks need to be deleted. Following this, the function BasePks_Curated() creates an updated data matrix based on the content of the “EICs_BasePeaks_Curated” directory. The curated data matrix (named “PksBpsCurated") is saved in a comma separated value file (.csv) within the working directory.

Additionally, MStractor outputs that can be used as confirmation of workflow progression are the printed EICs for all detected features, including both raw (step 4) and retention-time-corrected data (step 6); and data tables generated at steps 7, 8, and 9 that summarize detected feature information (feature intensity, *m/z*, and retention time).

#### 2.2.4. Results

MStractor results were evaluated by comparing the output obtained from processing the same dataset using XCMS Online. A detailed description of the workflow parameters is provided in the Materials and Methods section. Comparison of further data visualization and data analysis tools will be discussed in Section 2.2.5.

The XCMS Online results were returned in the form of a table; in total, 5656 features were detected. However, XCMS Online generated a data table that included all detected features, and did not provide the users with any tools for data curation or data reduction. Although very detailed, the result table contained redundant information (features belonging to the same peak group and, possibly, noise), and it might be difficult to interpret or use for further data analysis.

In this respect, XCMS Online’s output can be compared to the MStractor’s “PksAn” data matrix, which is generated after CAMERA annotation and is stored in the QC directory. The number of features listed in the “PksAn” data matrix amounted to 2312. This was significantly lower than the features detected via XCMS Online; however, this was expected since MStractor allows the definition of a minimum “integration threshold” value (set to 2000) and a “sensitivity” value (set to 0.7); both limit the inclusion of background noise.

**Figure 3:**
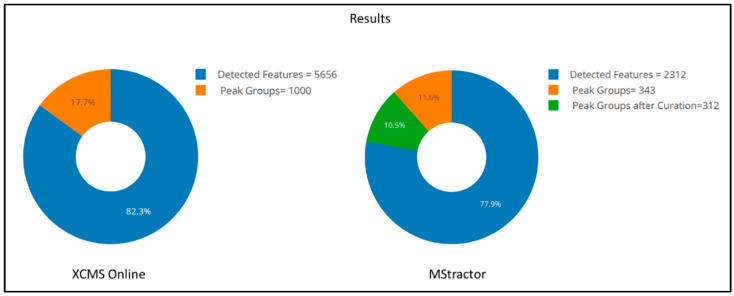
Result comparison for XCMS Online and MStractor.

Further data reduction and curation steps were subsequently performed in MStractor to rationalize the large amount of data generated. These steps included:

- Condensing peak groups by retaining only the most intense feature for each peak group (FilterDM()). This feature selection assumes that features in an assigned group belong to the same chemical entity. After filtering, the matrix was reduced to 343 peaks. The same data-reduction step was manually performed for comparison purposes on the XCMS Online dataset, which was reduced to 1000 features.
- Performing manual curation via CollectBP_EICs() and BasePks_Curated(). This step was aimed at removing background signals and peaks that were not well resolved (described in Section 2.2.3). During the curation step, 31 features were removed, and the final data table contained 312 features. This further curation step, however, was not available in XCMS Online, since EICs are not generated for all the features. The MStractor and XCMS Online results are summarized in Figure 3.
- Median normalization, which was carried out to minimize possible inter-run instrument variability.
- Calculation of descriptive statistics via the statsByClass() function that returned separate data tables containing average values, standard deviation, and coefficient of variation (%CV ) for each sample class (Treatment and Mix in the present dataset).

An extract of the result tables generated by XCMS Online and MStractor is reported in Tables S1–S4.

The structure of the result data table generated by MStractor also provides the user with additional information. As an example, instrument response is displayed for each sample, while XCMS Online only returns the average response among classes and the standard deviation. Conversely, descriptive statistics results (average, standard deviation and %CV) are calculated and stored separately using MStractor.

#### 2.2.5. Data Analysis and Visualization

Result visualization and data analysis provide quite different outputs in MStractor and XCMS Online. Each of the different tools are discussed in the following section.

**Figure 4:**
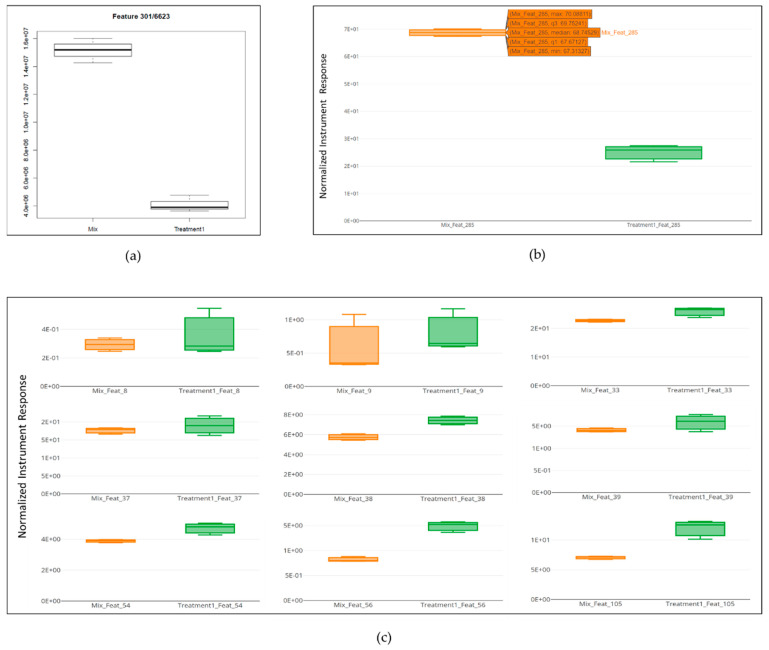
Box plots: (**a**) XCMS Online; (**b**) MStractor individual box plot with source data displayed upon hovering; (**c**) MStractor group box plots.

**Figure 5:**
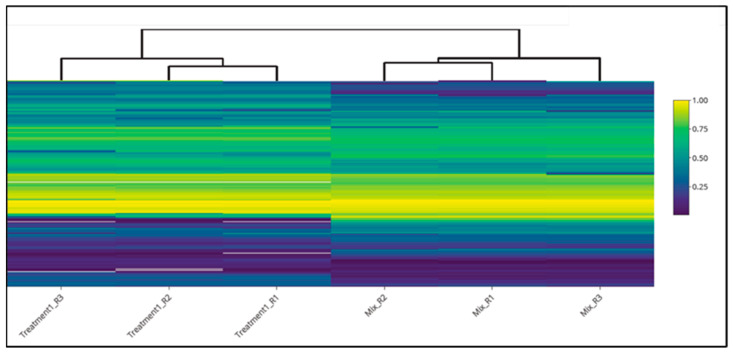
Heat map generated by MStractor.

**Figure 6:**
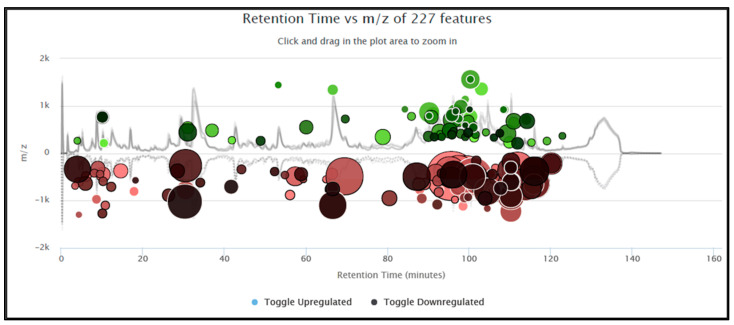
Interactive cloud plot generated by XCMS Online.

- Extracted Ion Chromatograms (EICs) and Box Plots
  EIC visualization and box plots are available in XCMS Online depending on the output of the *t*-test (displayed in the result table). If the feature result is not significant, the plot is not generated. This is quite limiting, as the user cannot visualize all the metabolites in the biological sample. On the contrary, MStractor does not perform a significance test, but provides extracted ion chromatograms and box plots for every feature. A *t*-test was not included among MStractor features because it was designed to be able to accommodate pairwise and multigroup comparisons without the need of selecting dedicated workflows, as required by XCMS Online. Extracted ion chromatogram plots are automatically generated at different stages of the workflow and stored in dedicated folders.
  In regard to box plots, MStractor provides more advanced visualization compared to XCMS Online. Using the bpSel() function, a dedicated GUI enables the user to select the classes to be represented in the box plot (an option that is particularly valuable in case of multigroup experiments). Both individual box plots and group box plot (Figure 4) visualizations are saved as .html files. This allows performing immediate visual comparisons of the analyte differences among sample classes. In addition, all the plots are interactive, allowing zooming in, as well as source-data display upon hovering.
- Heat map, Principal Component Analysis and Cloud Plot
  Heat maps generated in MStractor and XCMS Online are very similar, as both provide interactive visualization (Heatmaply package for MStractor [18]). However, the heat map in XCMS Online can only display a limited number of features (first 1000). Thanks to the data-reduction steps, the MStractor user is provided with a heat map for the whole dataset, avoiding a partial visualization. Using the present case study, the heatmap could not be generated in XCMS Online. This, however, could be related to the speed of the internet connection, rather than XCMS Online’s computational power. An example of the heat map produced in MStractor is reported in Figure 5.
  On the other hand, XCMS Online provides PCA and cloud plots. Specifically, interactive cloud plot functionality is useful, since it represents the feature fold change along the retention time domain as bubble plots. A number of interactive settings are also available to filter the results based on intensity, *m/z*, rt, p-value, and fold change. An example is reported in Figure 6. This type of feature is not available in MStractor.
- Library Search, Putative Identifications
  Library search and putative identifications represent another point of divergence between MStractor and XCMS Online.
  Within XCMS Online, parameters for putative identification are entered via the “Identification” tab, which allows users to define the mass tolerance and select possible adducts.
  Library search is performed via the METLIN database using the feature mass and the corresponding fragmentation pattern. On one hand, this option proves very valuable for datasets containing MS/MS data. On the other hand, when MS1 data are used (such as in the example dataset considered), the confidence of the putative identification performed might drop significantly, as it is based only on the accurate mass of the molecular ion and its isotopic pattern.
  In MStractor, the spectrum of each chemical entity is saved as a list of *m/z* vs. intensities that are stored as .msp files. This type of file can be uploaded in the NIST software and searched for matches against a given spectral library.
  Using GUIs, StoreRefFeat() and spectraFromScan() functions extract the raw spectra from a selected reference file, nistEntryFromScan() creates individual NIST compatible entries for each compound, while createSearchList() lists all compound spectra with unique identifiers in the .msp file. In this way, the library search can be performed for a single compound of interest or, alternatively, for all the compounds at once.

## 3. Materials and Methods

### 3.1. Samples and Sample Preparation

The sample set included in the package is for demonstration purposes and includes wine samples and a pooled biological mix. The wine samples include three biological replicates of a white wine, named “Treatment”, while the pooled biological sample used for instrument quality control (QC) was prepared by mixing equal aliquots of each sample and named “MIX”.

A 1 mL aliquot of each sample was transferred in a 2 mL amber HPLC screw-cap vial and kept at 4 °C prior to analysis. Three biological replicates for each wine and three analytical replicates of the MIX were analyzed by LC/MS. The samples were injected neat.

### 3.2. HPLC-QToF MS Analytical Platform

Samples were analyzed using an Agilent 1200SL HPLC (Agilent, Santa Clara, CA, USA) coupled to a high-resolution Bruker micrOTof QII (Bruker Daltonics, Karlsruhe, Germany).

The Agilent system was equipped with a degasser, an autosampler, a binary pump, and a column oven. Chromatography analysis was carried out using a reverse phase (RP) method adopting a Kinetex PFP column (150 mm × 2.1 mm ID, 2.6 μm particle size) manufactured by Phenomenex (Torrance, CA, USA). The column temperature was set at 30 °C.

The mobile phases were solvent A (0.1% formic acid, 0.5% methanol in MilliQ water) and solvent B (0.1% formic acid, 2% MilliQ water, 40% acetonitrile in methanol). All the solvents were gradient grade for liquid chromatography from Supelco (Bellefonte, PA, USA). The flow was held constant at 0.4 mL/min, and the gradient used started at 0% B; the percentage of solvent B was then increased to 1% at 25 min, 7.5% at 80 min, 60% at 125 min, and 90% at 130 min, and then held for 3 min. The column was washed and re-equilibrated from 133 to 147 min. The sample injection volume was 1 μL.

The mass spectrometer was operated in negative ESI mode using the following source conditions: temperature 200 °C, capillary voltage 3500 V, end plate offset −500 V, nebulizer pressure 2.0 Bar, dry gas flow 7.0 L/min.

The mass range acquired was from 50 to 1650 amu and the acquisition rate 0.5 Hz in MS1 mode.

The instrument was calibrated by infusing a 10 mM sodium formate solution at a rate of 100 μL/h. The calibrant was also injected at the beginning of each run to perform a post-run calibration.

### 3.3. Dataset Preparation

Prior to running the workflow, data were translated into the mzXML generic file format (other supported formats include CDF, mzData, mzML) using Proteo Wizard (Proteo Wizard, http://proteowizard.sourceforge.net/, accessed 13 April 2021).

The dataset used in this paper is freely available and can be downloaded with the package at https://github.com/MetabolomicsSA/MStractor, accessed 1 July 2021.

### 3.4. Hardware and Software

The example dataset was processed using R version 4.1.0 and RStudio Version 1.3.1093 on a 64-bit Windows 10 Pro operating system with 32 GB of RAM and an Intel(R) Core (TM) i7-8809G CPU @ 3.10GHz processor.

The same dataset was processed in parallel using XCMS Online (https://xcmsonline.scripps.edu, accessed 30 July 2021) using the “pairwise” job option.

Where possible, the same parameters were used for feature extraction. The full list is reported in Table 1. Besides the parameters listed in Table 1, XCMS Online also gives the option to perform pathway mapping. However, this option was not used because it goes beyond the scope of this paper.

## 4. Conclusions

The MStractor package was critically evaluated and compared to the existing XCMS Online workflow. The main advantages of MStractor are preliminary visual outputs to evaluate parameter settings, data QC at various processing stages through graphical output and data tables, data reduction that rationalize complex data, median normalization, and detailed result tables.

In this respect, MStractor provides a quick and efficient alternative for users that prefer to process their data in an offline environment, while still maintaining advanced data extraction, QC monitoring, and visualization tools, all within a single package.

MStractor is used routinely in our laboratory by multiple technical staff and scientists for effectively processing a broad range of data from untargeted metabolomics experiments with biomedical, agricultural, and food samples. By offering the possibility of a deeper control on parameter settings, MStractor has proven very valuable, not only for users with minimal coding skills, but also for experienced R users. Finally, being coded in R, the MStractor workflow can be easily adapted, extended, integrated, or enhanced with other existing applications.

## Figures and Tables

**Table 1:** Parameters used for XCMS Online and MStractor.

	XCMS Online	MStractor
	**Feature Detection CentWave**	
ppm	10	10
Min peak width (seconds)	10	10
Max peak width (seconds)	20	20
Signal-to-noise threshold	100	100
m/z difference	0.01	0.01
Integration method	1	1
Prefilter peaks	100	100
Prefilter intensity	750	750
Noise filter	None	Not applicable
Integration threshold	Not applicable	2000
Sensitivity	Not applicable	0.7
Fit Gaussian	Not applicable	FALSE
	**Retention Time Correction**	
Method	loess	loess
Extra peaks	1	1
Missing	3	3
Bw (seconds)	20	20
Mzwid	0.1	0.1
Minfrac	0.5	0.5
Span	0.6	0.6
Family	Gaussian	Gaussian
	**Alignment**	
Bw (seconds)	20	20
Minfrac	0.3	0.3
Mzwid	0.1	0.1
Minsamp	2	2
Max	50	50
	**Peak annotation**	
Sigma	Not applicable	6
Percentage of FHWM	Not applicable	1
Intensity	Not applicable	maxo
Max number of expected isotopes	Not applicable	4
ppm error	10	10
m/z absolute error	0.005	Not applicable
Group-correlation threshold	Not applicable	0.7
Intensity-correlation threshold	Not applicable	0.7
Correlation-threshold significance	Not applicable	0.1
	**Identification**	
ppm	10	Not applicable
adducts	[M − H]^−^, [M + FA-H]^−^	Not applicable

## Data Availability

The package and dataset described in this paper are available on GitHub https://github.com/MetabolomicsSA/MStractor/, accessed on 1 July 2021.

## Supplementary Materials

The following are available online at https://www.mdpi.com/article/10.3390/metabo11080492/s1: Table S1: MStractor raw result table extract, Table S2: MStractor normalized result table extract, Table S3: MStractor descriptive stats result extract, Table S4: XCMS Online result table extract, Figure S1: Workflow commands, Figure S2: Example of MStractor GUI for parameter input.
