# Supplementary material for: MStractor: R Workflow Package for Enhancing Metabolomics Data Pre-Processing and Visualization
Source: Metabolites. 2021 Jul 29;11(8):492. doi: 10.3390/metabo11080492 (PMC8398219; doi:10.3390/metabo11080492)

Supplementary Information

Table S1: Extract of the MStractor workflow result table (‘PksBpsCurated’)

Id	mz	mzmin	mzmax	rt	rtmin	rtmax	npeaks	Mix	Treatment1	ms_level	Mix_R1	Mix_R2	Mix_R3	Treatment1_R1	Treatment1_R2	Treatment1_R3	isotopes	adduct	pcgroup	RespMed
184	248.9607	248.9606	248.9608	14.1	13.5	14.6	6	3	3	1	79514	76798	74180	79184	76473	76738	[13][M]-		47	76768
216	272.9591	272.9588	272.9595	90.4	89.5	90.6	6	3	3	1	12465	11566	11666	6607	5014	9258			199	10412
183	248.9606	248.9603	249.0416	94.5	93.5	103.5	7	3	3	1	14081	13294	13320	16880	15490	10346			164	13700.5
83	197.808	197.8076	197.8083	102.4	102.2	102.5	3	1	2	1	8464	8472	8605	11400	4956	890			217	8468
19	149.0097	149.0093	149.01	106.5	105.5	106.6	6	3	3	1	6462	6663	6606	3189	3871	16758			166	6534
64	187.0423	187.0415	187.0427	124.6	123.5	124.6	6	3	3	1	5190	4543	4949	2688	3648	12726			193	4746
8	117.0195	117.0191	117.0199	153.9	152.8	154.7	4	2	2	1	2436	2955	2647	1989	2173	4911			298	2541.5
9	125.0248	125.0247	125.0251	215.1	211.4	261.0	5	2	3	1	3241	3065	9727	5217	8964	5328			766	5272.5
554	385.0236	385.0229	385.0243	220.9	220.5	221.4	6	3	3	1	17005	16492	16618	18987	25105	34520	[40][M]-		91	17996
33	169.0146	169.0143	169.015	261.1	260.5	261.4	6	3	3	1	219586	202016	204388	215307	208555	213596	[4][M]-		14	211075.5
1576	689.0824	689.0815	689.0836	271.4	271.0	272.8	6	3	3	1	51372	46741	48856	56926	53127	54888	[134][M]-		62	52249.5
973	495.1138	495.1129	495.1147	278.3	277.2	279.4	2	1	1	1	4032	3677	3843	2120	2177	2400			373	3038.5
1517	673.0876	673.0872	673.0881	293.4	293.1	294.9	6	3	3	1	101222	94086	95145	107601	95512	91835	[128][M]-		37	95328.5
505	369.0289	369.028	369.0293	313.0	311.4	313.5	6	3	3	1	215345	195878	203339	306884	308352	363012	[35][M]-		7	261114.5
54	183.0304	183.0302	183.0309	329.3	328.5	329.6	6	3	3	1	37352	33962	35811	38963	38768	38393	[5][M]-		82	37872.5
501	368.0983	368.0977	368.0987	344.8	343.4	345.7	4	3	1	1	3761	3418	3428	4922	5955	6749			313	4341.5
970	495.1134	495.1129	495.1142	369.7	369.3	371.1	6	3	3	1	7748	7246	7192	2736	2607	3111			281	5151.5
698	424.0263	424.0263	424.0263	415.9	415.9	415.9	1	0	1	1	1832	1330	1421	2304	2458	3265			491	2068
23	153.0561	153.0558	153.0566	428.1	427.4	429.5	5	2	3	1	15861	15498	15594	20494	19506	18731			139	17296
1931	867.2059	867.2059	867.2059	449.6	449.6	449.6	1	0	1	1	12540	10849	10976	7852	7119	7272	[181][M]-		198	9350.5
1519	673.0874	673.0866	673.0907	455.2	443.4	456.2	4	3	1	1	87351	81061	11476	83448	78534	72630	[129][M]-		45	79797.5
22	153.0199	153.0197	153.0204	479.9	479.4	480.4	6	3	3	1	36145	34251	32711	32759	30826	34714	[2][M]-		86	33505
688	421.1037	421.1032	421.1052	488.4	487.7	489.5	5	3	2	1	20362	18163	17641	7936	7631	9261			141	13451
37	175.0617	175.0614	175.0625	516.1	515.4	516.5	6	3	3	1	164033	159497	160827	131861	145864	194632			17	160162
39	176.0649	176.0649	176.0649	516.4	516.4	516.4	1	1	0	1	13512	12681	12616	11135	12368	15819			634	12648.5
1460	657.0926	657.0916	657.0943	524.4	522.4	525.6	6	3	3	1	426497	394609	402076	535640	510217	452758	[121][M]-		3	439627.5
2125	973.2024	973.2007	973.207	528.4	527.8	530.5	5	2	3	1	8015	7031	7177	2420	2652	2733			635	4882

Id: identifier assigned in the MStractor workflow; **mz**: accurate mass to charge ratio, **mzmin**: minimum m/z observed for the feature across the dataset, **mzmax**: max m/z observed for the feature across the dataset **rt**: retention time expressed in minutes, **rtmin**: minimum rt observed for the feature across the dataset, **rtmax**: maximum rt observed for the feature across the dataset, **npeaks**: number of peaks observed for a given feature in the dataset, **isotopes**: isotopes found in the pseudospectrum, **adducts**: adducts presents, **pcgroup**: id of the pseudospectra groups, **RespMed**: average response across the dataset. **Mix_R1, Mix_R2, Mix_R3, Treatment1_R1, Treatment1_R2, Treatment1_R3**: instrumental response detected in the samples.

Table S2: Extract of the MStractor workflow result table (Median normalized data table)

Id	mz	rt	Mix_R1	Mix_R2	Mix_R3	Treatment1_R1	Treatment1_R2	Treatment1_R3
184	248.9607	0.23	8.04	8.82	8.24	9.77	9.95	8.55
216	272.9591	1.51	1.26	1.33	1.30	0.82	0.65	1.03
183	248.9606	1.58	1.42	1.53	1.48	2.08	2.01	1.15
83	197.808	1.71	0.86	0.97	0.96	1.41	0.64	0.10
19	149.0097	1.78	0.65	0.77	0.73	0.39	0.50	1.87
64	187.0423	2.08	0.53	0.52	0.55	0.33	0.47	1.42
8	117.0195	2.57	0.25	0.34	0.29	0.25	0.28	0.55
9	125.0248	3.58	0.33	0.35	1.08	0.64	1.17	0.59
554	385.0236	3.68	1.72	1.89	1.85	2.34	3.27	3.85
33	169.0146	4.35	22.22	23.21	22.71	26.57	27.13	23.81
1576	689.0824	4.52	5.20	5.37	5.43	7.02	6.91	6.12
973	495.1138	4.64	0.41	0.42	0.43	0.26	0.28	0.27
1517	673.0876	4.89	10.24	10.81	10.57	13.28	12.42	10.24
505	369.0289	5.22	21.79	22.50	22.59	37.87	40.11	40.47
54	183.0304	5.49	3.78	3.90	3.98	4.81	5.04	4.28
501	368.0983	5.75	0.38	0.39	0.38	0.61	0.77	0.75
970	495.1134	6.16	0.78	0.83	0.80	0.34	0.34	0.35
698	424.0263	6.93	0.19	0.15	0.16	0.28	0.32	0.36
23	153.0561	7.13	1.60	1.78	1.73	2.53	2.54	2.09
1931	867.2059	7.49	1.27	1.25	1.22	0.97	0.93	0.81
1519	673.0874	7.59	8.84	9.31	1.28	10.30	10.22	8.10
22	153.0199	8	3.66	3.94	3.63	4.04	4.01	3.87
688	421.1037	8.14	2.06	2.09	1.96	0.98	0.99	1.03
37	175.0617	8.6	16.59	18.32	17.87	16.27	18.97	21.70
39	176.0649	8.61	1.37	1.46	1.40	1.37	1.61	1.76
1460	657.0926	8.74	43.15	45.34	44.68	66.10	66.37	50.47
2125	973.2024	8.81	0.81	0.81	0.80	0.30	0.34	0.30

Id: Feature identifier assigned in the MStractor workflow; **mz**: accurate mass to charge ratio **rt**: retention time expressed in minutes.
Mix_R1, Mix_R2, Mix_R3, Treatment1_R1, Treatment1_R2, Treatment1_R3: median normalized instrumental response detected in the samples.

Table S3: Extract of the MStractor descriptive statistics table (MIX)

mz	rt	Mix_R1	Mix_R2	Mix_R3	Average.Mix	St.Dev.Mix	CV.Mix
509.0745	106.02	0.24	0.23	0.26	0.24	0.01	5.50
739.2242	111.41	0.81	0.83	0.82	0.82	0.01	1.07
315.0518	116	39.91	37.98	38.30	38.73	1.03	2.67
528.3041	98.67	0.28	0.26	0.28	0.27	0.01	4.24
169.0146	4.35	22.22	23.21	22.71	22.71	0.50	2.19
317.0315	104.64	34.00	34.59	37.08	35.23	1.64	4.65
319.1309	89.91	0.25	0.27	0.26	0.26	0.01	4.98
321.1738	129.44	3.15	3.02	3.04	3.07	0.07	2.21
323.0785	101.78	0.79	0.82	0.83	0.81	0.02	2.32
325.0586	47.45	3.25	3.32	3.37	3.31	0.06	1.77
325.0911	35.4	3.71	3.93	3.89	3.84	0.12	3.06
326.0613	46.71	0.96	1.00	1.02	0.99	0.03	3.31
741.2223	99.13	0.18	0.18	0.19	0.18	0.00	2.63
175.0617	8.6	16.59	18.32	17.87	17.60	0.90	5.10
326.1723	88.31	0.32	0.32	0.30	0.31	0.01	4.16
509.129	11.07	0.88	0.86	0.87	0.87	0.01	0.91
531.0754	86.71	0.33	0.33	0.34	0.33	0.01	2.92
175.0617	10.02	5.45	6.08	5.73	5.75	0.32	5.49
176.0649	8.61	1.37	1.46	1.40	1.41	0.05	3.22
328.2218	111.02	1.17	1.21	1.22	1.20	0.03	2.27
329.2343	113.83	12.37	12.79	12.71	12.62	0.23	1.80
329.2323	111.18	0.87	0.90	0.89	0.89	0.01	1.51
329.2339	116.57	0.89	0.89	0.89	0.89	0.00	0.16
751.1885	99.55	1.97	1.97	2.00	1.98	0.02	0.82
331.0471	111.29	8.97	9.11	8.67	8.92	0.22	2.52
331.103	10.72	1.04	1.10	0.75	0.96	0.18	18.99
331.2432	114.16	0.31	0.50	0.40	0.41	0.09	23.22
333.0621	99.71	0.93	0.95	0.99	0.95	0.03	3.53
531.1162	106.41	1.02	1.10	1.06	1.06	0.04	3.50

mz: accurate mass to charge ratio; **rt**: retention time expressed in minutes;
Mix_R1, Mix_R2, Mix_R3: median normalized instrumental response detected in the
Average Mix, St. Dev. Mix, CV Mix: descriptive stats calculated on the QC Mix replicates.

Table S4: Extract of the XCMS Online result table

featureidx	MSMS	fold	log2fold	tstat	pvalue	qvalue	updown	mzmed	mzmin	mzmax	rtmed	rtmin	rtmax	maxint	Treatment1_mean	Treatment1_sd	Mix_mean	Mix_sd	isotopes	adducts	peakgroup	name	METLIN_ MSMS
4146		1.1	0.1	0.9	0.42	0.25	UP	1185.719	1185.719	1185.736	0.20	0.20	0.24	1007	9548	1295	10453	1188	[441][M+1]-		46	M1186T0	n
3782		1.1	-0.2	-1.1	0.33	0.21	DOWN	1091.733	1091.732	1091.738	0.21	0.17	0.27	1080	12360	1193	11022	1666			46	M1092T0_1	n
2600		1.2	-0.3	-2.2	0.10	0.10	DOWN	1033.725	1033.719	1033.725	0.22	0.20	0.27	1560	20283	1473	16938	2177	[397][M+1]-		46	M1034T0	n
2830		1.2	-0.2	-1.9	0.14	0.12	DOWN	1101.711	1101.706	1101.714	0.22	0.20	0.27	1193	15723	1176	13586	1559	[419][M+1]-		46	M1102T0	n
2349		1.3	-0.3	-2.9	0.07	0.08	DOWN	1058.738	1058.729	1058.747	0.22	0.20	0.24	1012	12503	569	9872	1442	[406][M+1]2-		46	M1059T0	n
2682		1.2	-0.2	-2.4	0.11	0.10	DOWN	1169.693	1169.686	1169.699	0.22	0.21	0.24	798	10250	911	8909	334	[435][M+1]-		46	M1170T0	n
4768		1.1	-0.1	-0.5	0.64	0.33	DOWN	1236.692	1236.686	1236.698	0.22	0.21	0.24	840	9122	1952	8498	505			46	M1237T0	n
2882		1.3	-0.3	-1.8	0.14	0.12	DOWN	966.7361	966.7326	966.7396	0.22	0.20	0.27	1296	15990	2033	12557	2524	[365][M+2]-		46	M967T0	n
3197		1.3	-0.4	-1.8	0.20	0.15	DOWN	1125.226	1125.223	1125.231	0.22	0.20	0.27	986	11459	385	8947	2342	[426][M]2-		46	M1125T0	n
3252		1.2	-0.3	-1.6	0.21	0.16	DOWN	899.7458	899.742	899.7459	0.22	0.20	0.27	1049	12983	1138	10554	2380	[330][M+3]-		46	M900T0	n
2387		1.2	-0.2	-2.4	0.08	0.08	DOWN	965.7359	965.7313	965.7419	0.23	0.20	0.27	1815	22875	1738	19359	1858	[365][M+1]-		46	M966T0	n
2565		1.2	-0.3	-2.2	0.10	0.09	DOWN	898.7486	898.7428	898.7544	0.23	0.20	0.27	1324	16247	1501	13342	1749	[330][M+2]-		46	M899T0	n
3098		1.2	-0.2	-1.7	0.18	0.14	DOWN	844.3024	844.299	844.3076	0.23	0.20	0.27	1466	16106	1922	13925	1141	[305][M+1]2-		46	M844T0_2	n
3811		1.1	-0.1	-1.1	0.34	0.22	DOWN	198.9228	198.9222	198.9231	0.23	0.20	0.27	5336	78852	5928	74240	4022			46	M199T0	n
3877		1.1	-0.1	-1.2	0.35	0.22	DOWN	1151.24	1151.23	1151.241	0.23	0.20	0.27	1259	12771	1401	11788	307	[430][M+3]2-		46	M1151T0_2	n
1782		1.2	0.3	3.3	0.03	0.04	UP	1320.697	1320.692	1320.71	0.23	0.17	0.27	1085	9998	773	12185	831			46	M1321T0	n
2198		1.1	-0.2	-2.8	0.06	0.06	DOWN	949.7883	949.7851	949.7895	0.23	0.20	0.27	992	11135	467	9777	702	[356][M+4]-		46	M950T0	n
3287		1.1	0.2	1.5	0.22	0.17	UP	998.8158	998.8069	998.8195	0.23	0.20	0.27	1103	10289	1297	11669	993	[382][M+2]-		46	M999T0	n
3649		1.1	0.1	1.3	0.30	0.20	UP	1065.305	1065.304	1065.309	0.23	0.20	0.27	1285	11402	1494	12599	363			46	M1065T0_2	n
3925		1.1	-0.2	-1.0	0.36	0.22	DOWN	1084.253	1084.248	1084.255	0.23	0.20	0.27	1096	11651	1502	10199	1891	[413][M+5]2-		46	M1084T0_2	n
4230		1.1	-0.1	-0.8	0.45	0.26	DOWN	1185.232	1185.223	1185.239	0.23	0.20	0.27	1158	11911	1634	10912	1230			46	M1185T0_2	n
4300		1.1	0.2	0.8	0.47	0.27	UP	1336.729	1336.722	1336.74	0.23	0.20	0.27	1223	10378	2332	11850	2160			46	M1337T0	n
4556		1.1	-0.1	-0.6	0.56	0.30	DOWN	1150.241	1150.24	1150.246	0.23	0.20	0.27	1171	11443	1764	10605	1425	[430][M+1]2-		46	M1150T0_2	n
4878		1.0	0.0	-0.5	0.68	0.34	DOWN	845.7979	845.7978	845.8048	0.23	0.20	0.27	1205	13443	137	13032	1498	[305][M+4]2-		46	M846T0_1	n
5351		1.0	0.0	-0.2	0.88	0.40	DOWN	1302.728	1302.72	1302.735	0.23	0.19	0.27	1126	10297	1224	9975	3122			46	M1303T0	n
5421		1.0	0.0	-0.1	0.91	0.41	DOWN	1142.766	1142.761	1142.769	0.23	0.20	0.27	1191	10543	2607	10340	768	[428][M+1]2-		46	M1143T0	n
2897		1.1	-0.2	-1.8	0.15	0.12	DOWN	897.75	897.745	897.752	0.23	0.20	0.27	1804	22395	1799	19658	1915	[330][M+1]-		46	M898T0	n
3771		1.1	-0.2	-1.1	0.32	0.21	DOWN	829.7597	829.7589	829.7605	0.23	0.20	0.27	1737	22214	2985	19661	2550	[303][M+1]-		46	M830T0	n
5653		1.0	0.0	0.0	1.00	0.43	UP	813.8131	813.8077	813.8163	0.23	0.20	0.27	1018	11388	1375	11390	1366	[296][M+4]-		46	M814T0	n
2178		1.5	-0.5	-2.9	0.06	0.06	DOWN	990.7538	990.7503	990.757	0.23	0.20	0.24	1071	12870	1301	8859	2052	[380][M+1]2-		46	M991T0	n
4607		1.1	-0.2	-0.6	0.58	0.31	DOWN	913.7849	913.7755	913.7857	0.23	0.21	0.27	1634	17062	4159	15353	2341	[337][M+4]2-		46	M914T0_1	n

featureidx: feature identifier number; MSMS: ms/ms data, **fold** and **log2 fold**: foldchange and log2 fold change, **p-value, q value**: t test parameters, **updown**:whether the feature is up or down regulated, **mzmed**: average accurate mass to charge ratio, **mzmin**: minimum m/z observed for the feature across the dataset, **mzmax**: max m/z observed for the feature across the dataset **rt**: average retention time expressed in minutes, **rtmin**: minimum rt observed for the feature across the dataset, **rtmax**: maximum rt observed for the feature across the dataset, **npeaks**: number of peaks observed for a given feature in the dataset, **isotopes**: isotopes found in the pseudospectrum, **adducts**: adducts presents, **pcgroup**: id of the pseudospectra groups, **RespMed**:average response across the dataset.
Treatment1_mean, Mix_mean, Mix_sd: respectively, average instrumental response and standard deviation for the 2 sample classes considered. **Isotopes**: isotopes found in the pseudospectrum, **adducts**: adducts presents, **pcgroup**: id of the pseudospectra groups, **name**: entry identifier for Metlin library match, **METLIN_MSMS**: whether ms/ms library entries were used

Supplementary Information

Figure S1: MStractor workflow, sequence of functions with arguments

```
# Load library
library(MStractor)

# Acquisition parameters
Project()
LoadData()
DefineClassAttributes(ClassType)
ChromParam()
MassSpecParam()
PeakPickingParam()

#Explore reference files
exploreRefs()
refTic(x_refs)
get100(x_refs)

#Perfrom peak picking
peakPickGroup()

#Print raw EICs  and TICs
OverlaidTICs(xdata, 'raw')
printEICs(xdata, 'raw')

#Retention time alignment
RTalign(xdata,'loess')
OverlaidTICs(xdata, 'aligned')
pdp <- PeakDensityParam(sampleGroups = xdata$sample_group, minFraction =
0.3, bw = 20)
xdata <- groupChromPeaks(xdata, param = pdp)
xfilled <- fillChromPeaks(xdata, param =(FillChromPeaksParam(ppm = 10,
expandMz = 0.5)))

#Print aligned EICs  and TICs
OverlaidTICs(xdata, 'aligned')
printEICs(xfilled, 'filled')

#Create summary datamatrix
CreateDM(xfilled,'maxo')
xsetConvert(xfilled)
sampnames(xset)<-spn

#CAMERA
autoCamera(xsFilled)
FilterDM(PksAn, xset)
CollectBP_EICs(BasePks)
BasePks_Curated(BasePks)

#Descriptive statistics and data visualization
MedianNormalize(BasePksCur)
StatsByClass(ClassType)
bpSel()
Boxplot(NormalizedMatrix, bpSelection, "linear")

#Create spectra list for library search
storeRefFeat()
spectraFromScan(RefFeat)
nistEntryFromScan(spectralist, RefFeatScan)
createSearchList("spectralist")
```

Figure S2: MStractor GUI used for entering integration threshold value

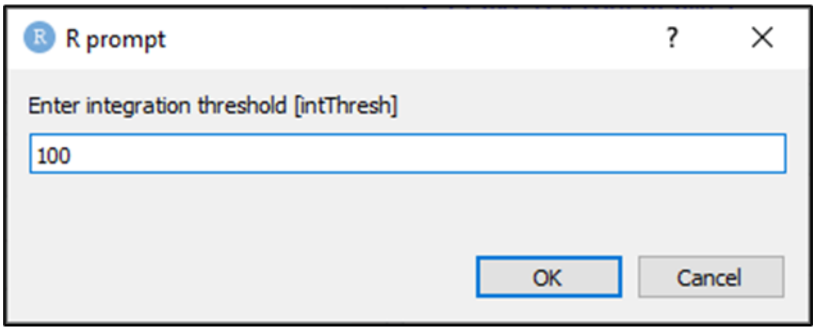

Supplement: Supplementary file 1 [file metabolites-11-00492-s001.zip › metabolites-1240748-supplementary.pdf]
